# Admittance of Atomic and Molecular Junctions and Their Signal Transmission

**DOI:** 10.3390/mi9070320

**Published:** 2018-06-25

**Authors:** Akira Sakai

**Affiliations:** Graduate School of Engineering, Kyoto University, Kyoto 6158540, Japan; sakai.akira.57a@st.kyoto-u.ac.jp

**Keywords:** atom-sized contacts of metals, single-atom contacts, molecular junctions, signal transmission, conductance, admittance

## Abstract

Atom-sized contacts of metals are usually characterized by their direct current (DC) conductance. However, when atom-sized contacts are used as device interconnects and transmit high frequency signals or fast pulses, the most critical parameter is not their DC conductance but their admittance Y(ω), in particular its imaginary part ImY(ω). In this article, I will present a brief survey of theoretical and experimental results on the magnitude of Y(ω) for atom-sized contacts of metals. Theoretical contact models are first described and followed by numerical evaluation of ImY(ω) based on these models. As for experiments on Y(ω), previous experiments conducted under time-varying biases are surveyed, and then the results of direct signal transmission through atom-sized contacts are discussed. Both theoretical and experimental results indicate that ImY(ω) is negligibly small for typical atom-sized contacts for signal frequencies up to 1 GHz.

## 1. Introduction

A never-ending challenge on downsizing electronic devices is nearly coming to accomplish its ultimate goal, realizing a device consisting of only one or a couple of atoms [1]. In such ultrasmall devices, connections to them must also be the size of atoms, and signal transmission through these atom-sized interconnects will become a vital issue for designing and operating atomic-scale devices. When a contact between two electrodes shrinks to one- or two-atoms wide, the contact size falls below the electron mean free path and the electronic conduction becomes ballistic. The conductance of a ballistic contact is given by the Landauer-Büttiker formula [2] which dictates $G=G_{0}\sum_{i}\tau_{i}$, where $G_{0}\equiv 2e^{2}/h$ is the quantum unit of conductance and $\tau_{i}$ stands for the electron transmission probability of the *i*-th conductance channel (conductance channels are eigenstates of a transmission matrix that connects the incoming and outgoing wave functions of an electron passing through a contact). Number of conductance channels that contribute to the conductance and their transmission differs for different elements and contact sizes. When a contact consists of only one atom of monovalent noble metals such as Au, Ag, and Cu, it has only one dominant conductance channel that shows a nearly perfect transmission τ∼1. As a result, single-atom contacts of these metals show a conductance $G∼1G_{0}$ as confirmed by numerous experiments [3,4].

This universal and material-independent conductance of single-atom contacts of Au, Ag, and Cu makes them quite useful as atomic switches [5,6,7,8]. Fast switching at 1 MHz has been demonstrated for Ag atomic switches [6], and mass-integration of single-atom switches to produce gate arrays have already been accomplished [9,10,11]. Conductance switching of Au atom-sized contacts have also been considered as consisting a memory operation of atomic devices [12].

**Figure 1:**
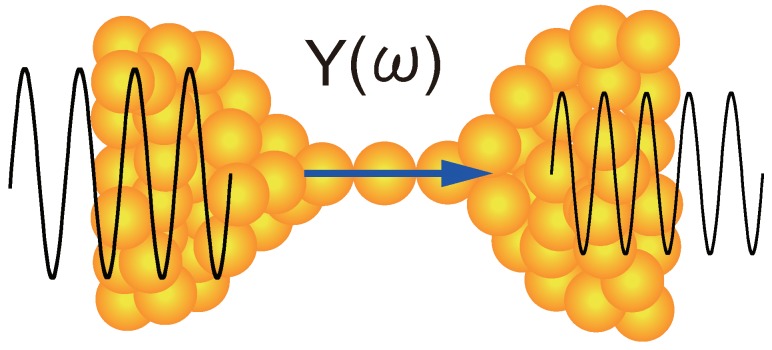
Transmission of a high-frequency signal thorough a single-atom contact.

These past device applications of atom-sized contacts are primarily concerned with conductance switching but not with their signal transmission. When using atom-sized contacts as interconnects, one has to first clear up such questions: does the admittance Y(ω) (or impedance Z(ω)=1/Y(ω)) of atom-sized contacts of metals remain unchanged when ω increases from direct current (DC) to 1 GHz? Do they transmit high-frequency signals or fast pulses as pure resistors even when their sizes shrink to a single atom (Figure 1)? Intuitively, answers to these questions appear obvious; a strong coupling between electrodes and contact atoms in metal contacts would arise no significant phase delay, positive or negative, between input and output signals and hence ImY(ω)∼0. Nevertheless, from a practical point of view, it should be necessary to quantitatively estimate the contact admittance and, if it is small, establish its smallness through measurements. In this article, I will discuss the admittance and the signal transmission of atom-sized contacts of metals. From a viewpoint of device applications, I will focus my discussion mainly on those contacts that show τ∼1 and $G∼1G_{0}$ (e.g., single-atom contacts of Au, Ag, and Cu). In Section 2, I will take up two simple contact models and try to make an order-of-magnitude estimation of the admittance of atom-sized contacts of metals. Experimental verification of the theoretical estimation will be mentioned in Section 3.

Except in Section 4, discussions will be made on atom-sized contacts of metals but not on molecular junctions. This is because molecules are poor conductors and unsuitable for signal transmission. Molecular junctions should be most valuable as functional elements but not as interconnects. It is, however, noted that carbon nanotubes and graphene nanoribbons exhibit high carrier mobility and have already been enjoying various applications as high-frequency devices and components [13]. This topic deserves a separate review.

## 2. Theoretical Calculations of Contact Admittance

### 2.1. Microscopic Calculations

By virtue of recent advancements in the method of electronic-structure calculation, a variety of toolkits, such as non-equilibrium Green function method (NEGF, see for example Reference [2]) and the real-space finite-difference method [14], are now available for theoretically tackling the problem of electron transmission thorough atom-sized contacts. In these calculations, one first constructs an atomistic contact model and then calculates the transmission of an electron wavefunction from one electrode to another. Then, the conductance can be calculated from the transmission probability using Landauer-Büttiker formula. However, these calculations implicitly assume a constant bias and hence yield a DC conductance. For obtaining the contact admittance, one has to extend the calculation scheme so as to take into account the time-variation of the bias voltage. Such an extension of NEGF has already been made and applied to study the admittance of various systems such as resonant tunneling systems [15,16,17], mesoscopic waveguides [18], and quantum point contacts of two-dimensional electron gas [19]. Applications to atomistic systems have been made primarily on carbon nanostructures, e.g., nanotubes [20,21,22,23,24] and graphene nanoribbons [25]. However, atom-sized contacts of metals have been left outside the target of these microscopic admittance calculations, presumably because of the intuitive smallness of their admittance. For evaluating the admittance of atom-sized contacts, one therefore has to rely on simple model calculations. In the following subsections, I will start with a classical contact model and then introduce two contact models that focus on different aspects of the atom-sized contacts for estimating the theoretical contact admittance.

### 2.2. Classical Radio-Controlled (RC) Model

**Figure 2:**
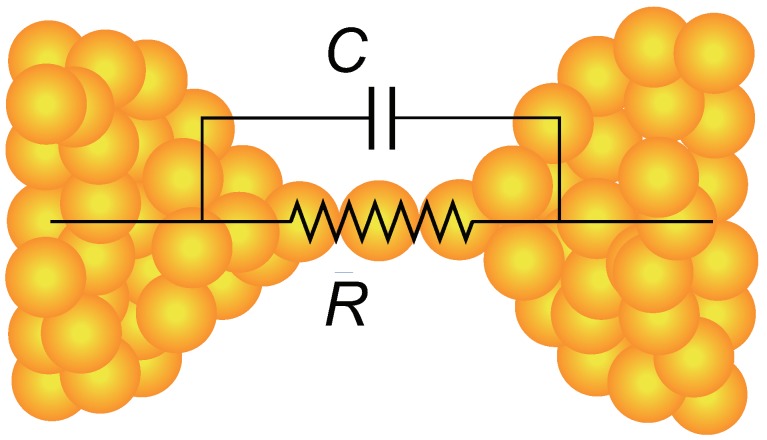
Classical resistor-capacitor (RC) model of a single-atom contact.

A contact model widely used in electronics is to represent a contact as a resistor-capacitor (RC) circuit composed of a resistor *R* and a capacitor *C* as shown in Figure 2. The contact admittance Y(ω) in this model can be written as Y(ω)=1/R+iωC. For ordinary (macroscopic) contacts, meanings of *R* and *C* are clear; *R* is a contact resistance and *C* measures any capacitive contributions that shunt the resistor. However, when the contact size becomes comparable to that of atoms, the definition of *R* and *C* becomes less obvious. The resistance *R* bears little problem; it can be interpreted as 1/G of the contact conductance *G*, even though *G* is now a non-classical (ballistic) conductance. For *C*, it can be naturally considered as a capacitance between contact electrodes, as illustrated in Figure 2. Though this interpretation appears plausible, some precautions must be made. First, this interpretation assumes that the atom link (the *R* part of the contact in Figure 2) makes no capacitive contributions. Theoretically, a classical resistor has no reactance, but it appears non-trivial whether or not the same is true for ballistic conductors. There remains a possibility that a ballistic contact shows a non-negligible reactance (or susceptance) and causes non-zero phase difference between voltage and current. Another point is the capacitor size. Because of the field concentration, the capacitive contribution mainly comes from the “base” (or “stem”) part of the contact. As a result, the effective capacitor size should be the size of atoms. The capacitance of such atomic-scale capacitors should be different from that of macroscopic capacitors and requires a non-classical approach for its understanding.

I will discuss these two problems separately. In the next section, I will describe the single-level model which focuses on the electron transmission through the atomic link and neglects all contributions from the base part of the contact. This model provides us information on the admittance of the atom link. In Section 2.4, the mesoscopic capacitor model tells us how the contact admittance deviates from the classical behavior when the contact (including both the atom link and the base part) shrinks to the size of atoms.

### 2.3. Single-Level Model

**Figure 3:**
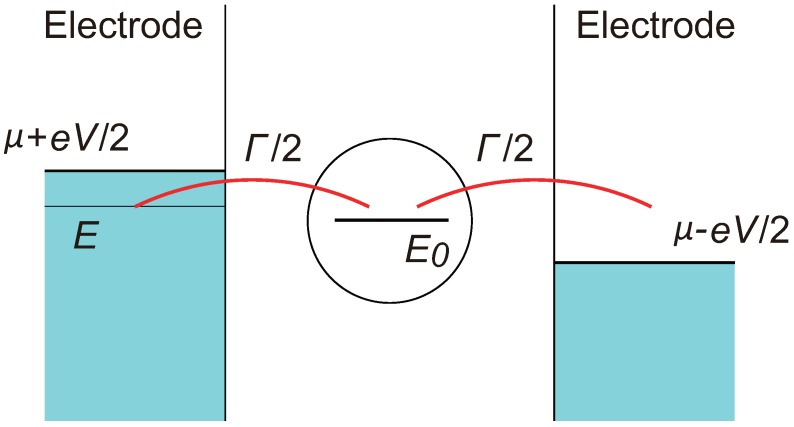
Schematic of the single-level model. A bridging atom is represented by a single energy level coupled with left and right electrodes. μ is the chemical potential of electrodes when the contact is in equilibrium. The bias *V* is time varying in the alternating current (AC) conduction of electrons. The transmission probability of an electron of energy *E* is given by Equation (1) in the text.

In the single-level model, a contact is represented by a single electronic level which couples with right and left electrodes, as illustrated in Figure 3. The electron transmission through this contact is determined by two parameters, the coupling strength of the single level with the right and left electrodes, denoted by $\Gamma_{R}$ and $\Gamma_{L}$, respectively, and the energy of the single level $E_{0}$. For simplicity, I assume here a symmetric contact and put $\Gamma_{R}=\Gamma_{L}\equiv \Gamma /2$. In Figure 3, the transmission probability of an electron of energy *E* can be expressed by a Lorentzian function and becomes maximum when $E=E_{0}$.

(1) $$T\left(E\right)=\frac{\Gamma^{2}}{{\left(E-E_{0}\right)}^{2}+\Gamma^{2}}$$

In the single-level model, a bridging atom(s) of an atom-sized contact (or a molecule of a single-molecular junction) is replaced just by a single energy level. Though this appears a large oversimplification, the model enjoys a wide applicability; by tuning the magnitude of Γ, one can emulate a variety of junctions [26], from almost insulating molecular junctions with Γ∼0 to highly conductive junctions of metal atoms where Γ→∞. The model can also properly describe non-linear *I*–*V* characteristics of various molecular junctions [4,27]. These successes of the single-level model come from the fact that, in typical atomic and molecular junctions, only one energy level among many levels of the bridging atom/molecule gives a dominant contribution to electron transmission. In molecular junctions, that is usually the Lowest Unoccupied Molecular Orbital (LUMO) or Highest Occupied Molecular Orbital (HOMO) level of the molecule and in the single-atom contacts of Au, Ag, and Cu, the single *s*-like eigenchannel leads to their $1G_{0}$ conductance.

The single-level model shown in Figure 3 can also be viewed as a double-barrier junction, or a resonant tunneling system, where Γ measures the barrier transmission. As mentioned before, the double barrier junction has been studied using NEGF [15,16,17] and its admittance has been discussed. I, however, adopt here the work of Fu and Dudley [28] because they have obtained an admittance formula that can be easily evaluated. They studied a linear response of a double-barrier junction against an alternating current (AC) input and derived the following set of equations for the real and imaginary parts of the junction admittance Y(ω),

(2) ReY(ω)=G0Γ4ℏωarctan(E0−μ)+ℏωΓ−arctan(E0−μ)−ℏωΓ

(3) ImY(ω)=−G0Γ8ℏωln[(E0−μ+ℏω)2+Γ2][(E0−μ−ℏω)2+Γ2]((E0−μ)2+Γ2)2

where μ is the chemical potential of electrodes when the contact is in equilibrium. As will be shown in the next section, the ω of “high-frequency” signals actually reside in a low frequency region in a sense that ℏω/Γ≪1. In this region, ReY(ω) and ImY(ω) can be approximated as (up to the first order in ℏω/Γ),

(4) ReY(ω)=τG0

(5) ImY(ω)=G04ℏωΓτ(1−2τ)

where $\tau =\Gamma^{2}/\left({\left(E_{0}-\mu \right)}^{2}+\Gamma^{2}\right)$. In Equation (4), ReY(ω) becomes a constant and ReY(0)=τG0 at ω=0. This result correctly reproduces the Landauer-Büttiker formula, and τ corresponds to the DC transmission probability of the contact. On the other hand, ImY(ω) in Equation (5) is linear in ω so that ImY(0)=0. Thus, as expected, the contact behaves as a pure resistor in the DC limit. The coefficient of ω in Equation (5) changes sign depending on the magnitude of τ. When τ>1/2, the coefficient is negative and the contact becomes inductive (in a sense that ImY<0). Conversely if τ<1/2, then ImY>0 and the contact shows a capacitive response. For transparent contacts with τ=1, Equations (4) and (5) become,

(6) ReY(ω)=G0

(7) ImY(ω)=−G04ℏωΓ

At another extreme of τ=0 (Γ=0), there is no coupling between electrodes (and hence no electron transmission) and Equations (2) and (3) naturally lead to Y(ω)=0.

### 2.4. Mesoscopic Capacitor Model

**Figure 4:**
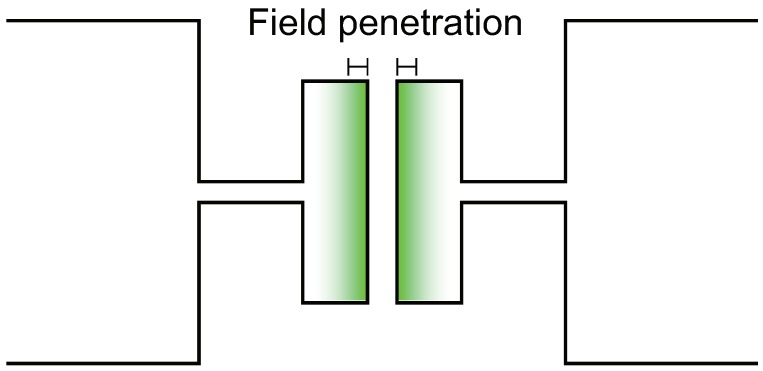
Schematic of the mesoscopic capacitor model. The color gradation schematically represents the field penetration mentioned in the text.

Another contact model that provides a calculable theoretical admittance is the mesoscopic capacitor model proposed and developed by Büttiker and coworkers [29,30,31,32]. This model starts with a mesoscopic capacitor, as schematically depicted in Figure 4.

Unlike a classical capacitor where all induced charges reside on the electrode surface, the mesoscopic capacitor can accommodate a finite amount of charges on its electrode surface determined by their surface density of states. As a result, the electric field slightly penetrates into the electrodes as illustrated in the figure. For metal electrodes, this field penetration depth, or the screening length, is the order of atomic distances and can be neglected for macroscopic capacitors. However, for mesoscopic capacitors, the field penetration becomes non-negligible and gives an additional capacitance in series with their classical (geometrical) capacitance $C_{0}$.

(8) $$\frac{1}{C}=\frac{1}{C_{0}}+\frac{2}{D}$$

This *C* is often referred to as the mesoscopic capacitance. The correction term *D* is related to the electrode surface density of states dN/dE through $D=e^{2}\left(dN/dE\right)$. Christen and Büttiker [32] further extended the model by allowing electron transmission between electrodes. The capacitor then becomes a “leaky” mesoscopic capacitor and its admittance can be written as (up to the first order in ω),

(9) ReY(ω)=τG0

(10) ImY(ω)=ω(1−τ)C−τ2D4

where *C* and *D* stand for the mesoscopic capacitance and its quantum correction, respectively, as before and τ is the electron transmission probability. In the DC limit ω→0, Equations (9) and (10) become ReY(0)=τG0 and ImY(0)=0, respectively, in agreement with the results obtained in the single-level model. For a transparent contact with τ=1, the mesoscopic capacitor model predicts,

(12) ReY(ω)=G0

(11) ImY(ω)=−ωD4

Comparison of these expressions with Equations (6) and (7) shows that two models yield the same ReY(ω) and similar ImY(ω). However, despite their formal similarity, ImY(ω)s in Equations (7) and (12) show different dependence on dN/dE and represent different aspects of the contact admittance. In the single-level model which focuses on the atomic link part in Figure 2, the increase in dN/dE tends to strengthen the coupling between the link atoms, making the link more like a pure resistor. On the other hand, in the mesoscopic capacitor model, the increase in dN/dE extends the charge-storage capacity of the contact electrodes and increases the contact capacitance. The two models also predict different results at the limit of no transmission τ=0; ImY(ω)=0 in the single-level model whereas ImY(ω)=ωC in the mesoscopic capacitor model. This discrepancy again reflects the difference between two models, which is that the capacitive coupling between electrodes, which exists even at τ=0, is not considered in the single-level model where the electrode couples with each other electrode only thorough the single level.

### 2.5. Theoretical Estimation of the Admittance of Atom-Sized Contacts

As noted before, signal frequencies used in device applications (up to a few GHz) correspond to low frequencies and in the low-frequency limit, the theoretical admittance can be written as Equations (4) and (5) and Equations (9) and (10) in the single-level model and in the mesoscopic capacitor model, respectively. Specifically, for single-atom contacts of Au, Ag, and Cu which would be of practical importance, τ∼1 and the admittance becomes Equations (6) and (7) and Equations (11) and (12). Both models yield the same ReY(ω)=G0. On the other hand, ImY(ω)=−(G0/4)(ℏω/Γ) in the single-level model, while ImY(ω)=−Dω/4 in the mesoscopic capacitor model. For quantitatively estimating the admittance, one therefore has to know the magnitude of coefficients Γ and *D* appearing in these expressions.

Concerning the coupling strength Γ, there is little experimental information on its magnitude even though the single-level model has been employed to quantitatively fit the results of shot noise measurements on Au single-atom contacts [33] (model parameters normalized by Γ were numerically evaluated but not Γ itself). For single-atom contacts of Au, Ag, and Cu, however, a crude estimation of Γ can be performed. First, according to Avriller and Levy Yeyati [34], $\Gamma ∼t^{2}\left(dN/dE\right)$ where *t* is the hopping integral between the contact atom and the electrodes and dN/dE is the electrode density of states. In the case of monovalent metals, the relevant hopping integral would be the one between *s* states and t∼(0.1−1) eV [35]. Combining these results suggest that Γ should be of the order of 0.1–1 eV. This leads to ImY(ω)=−(G0/4)(ℏω/Γ)∼−(0.01−0.1) nS at ω=1 GHz. Thus, ImY(ω) is negligibly small in the radio frequency (RF) region [36]. This smallness of ℏω/Γ also justifies the use of low-frequency approximation to Y(ω).

Different from Γ, there have been theoretical and experimental clues on the magnitude of *D* in Equation (12). Wang et al. [37] theoretically investigated the AC response of Al single-atom chains connecting jellium electrodes. They calculated the density of states dN/dE of the system and obtained the emittance (coefficient of ω in susceptance and corresponds to −D/4 in Equation (12)) for Al chains consisting 1–4 atoms. For all Al chains, the magnitude of the emittance was found less than ∼10 a.u. or ≲0.03 aF. Then, the susceptance becomes ImY(ω)≲0.03 nS at ω=1 GHz.

Experimental information on *D* comes from an observation of the Coulomb gap appearing in *I*–*V* characteristics of an scanning tunneling microscopy (STM) tip-sample junction. Hou et al. [38] measured *I*–*V* characteristics of a tip-Au-cluster junction and examined the Coulomb gap as a function of the tip-cluster distance *d*. From the data, they deduced the *d* dependence of the tip-cluster capacitance. The capacitance first exhibits a classical behavior and increases as 1/d when *d* decreases. Then, the capacitance exhibits a peak around d=0.15 nm and decreases with further reducing *d*. The observed non-classical behavior of the tip-sample capacitance can be understood by considering the tip-sample junction as a tunneling mesoscopic capacitor. For such capacitors, their mesoscopic capacitance slightly differs from Equation (10) due to non-zero transmission and can be written as,

(13) $$\frac{1-e^{-kd}}{C}=\frac{1}{C_{0}}+\frac{2}{D}$$

where *k* represents a decay constant. At large *d*, $C_{0}$ dominates the capacitance and *C* increases as ∼1/*d* as *d* decreases. On the other hand, at small *d*, the factor $1-e^{-kd}$ starts to suppress the increase in *C* and eventually *C* decreases with *d* as observed in experiment by Hou et al. They obtained C∼0.3 aF at their smallest distance d=0.13 nm. Assuming $(1-e^{-kd})/C∼2/D$ at this distance, the magnitude of *D* can be estimated as D∼0.6 aF using the reported decay constant $k=22.7\;{\mathrm{nm}}^{-1}$. Then, from Equation (14), the susceptance becomes ImY(ω)∼0.15 nS at ω=1 GHz. The magnitude of ImY(ω) in the mesoscopic capacitor model is thus comparable with that in the single-level model and equally negligible in the RF region.

It should be noted that the observed tip-sample capacitance ∼0.3 aF at the smallest distance is 10 times larger than the theoretical capacitance ∼0.03 aF calculated for Al atomic chains [37] and Al atomic junctions [39]. This is presumably due to the simplified atomic geometry of the junctions assumed in the capacitance calculations. In addition, Hou et al. found discrepancies between Equation (13) and the observed *d* dependence of the capacitance. They suggested that *D* might not be a constant but *d* dependent. Orders-of-magnitude variation of *D*, however, appears unlikely and the smallness of ImY(ω) in the RF region would remain unaltered. The numerical estimations of ImY(ω) mentioned above show that both the single-level model and the mesoscopic capacitor model consistently predict negligible ImY(ω) up to ω=1 GHz for transparent (τ=1) contacts such as the single-atom contacts of Au, Ag, and Cu. If we use the terms of the classical RC model shown in Figure 2, these single-atom contacts have negligible *C* and their *R* contains no appreciable reactance. Experimental verification of this conclusion will be described in the next section.

## 3. Experiments on the Admittance of Atom-Sized Contacts of Metals

Before mentioning about experiments on atom-sized metal contacts, it should be noted that these contacts are usually specified by their DC conductance in unit of $G_{0}$, e.g., $1G_{0}$ contacts, $3G_{0}$ contacts, and so on. This convention is adopted because one can measure the conductance of an atom-sized contact but cannot know its size unless one makes a direct observation with high-resolution-transmission-electron-microscopy [40,41,42,43]. In addition, there is no reliable size-conductance relationship that covers down to a single atom and works for many metals. For Au, Ag, and Cu, it is widely accepted that their $1G_{0}$ contacts are single-atom contacts. Though there remains a possibility that some multiple-atom contacts may happen to show the $1G_{0}$ conductance, such a coincidence would be a rare event as indicated by previous simulation studies [44,45]. The experimental results obtained on the $1G_{0}$ contacts of Au, Ag, and Cu can thus be safely interpreted as those on their single-atom contacts. In this section, I follow this interpretation and identify the $1G_{0}$ contacts of Au and Cu as their single-atom contacts.

### 3.1. Experiments under Alternating Current (AC) or Time-Varying Biases

Experiments on atom-sized contacts of metal are usually carried out under a constant DC bias and time-varying biases are rarely used. This might come from the fact that atom-sized metal contacts of metals exhibit straight *I*–*V* curves [46,47] and exhibit no spectral features that require bias modulation for their detection. However, there have been a few experiments that employ modulated biases or fast bias ramps. For example, in the experiments exploring the current disruption of single-atom contacts [48,49,50], the bias voltage was ramped to a high value in a short time interval. The conductance of Au single-atom contacts was found unchanged during the bias ramp of ∼10 μs [50], and this observation indirectly suggests that the electron transmission should be unaffected by the bias variation for frequencies up to ∼0.3 MHz.

Another group of experiments using modulated biases is an inelastic transmission spectroscopy [27,51] where the second derivative of the *I*–*V* curve is measured with the lock-in detection technique. The modulation frequency is typically a few kHz or lower. At these low frequencies, no influence of bias modulation can be expected, and the experiments in fact showed no modulation-related anomalies. Bias modulation has also been used in the shot-noise experiments [33,52,53,54,55,56]. Natelson and his coworkers [53,54,55,56] conducted their noise measurements on Au atom-sized contacts under AC biases with frequencies up to 520 MHz. At such high frequencies, one usually has to worry about possible effects of contact admittance. However, their results are not much different from those [33] obtained at lower frequencies. Up to this time, no experiments on atom-sized contacts of metals, employing either ramped or modulated biases, have indicated any frequency dependent transport phenomena. These previous experiments, however, used time-varying biases only for some technical reasons and provided no direct information on the admittance. For verifying the theoretical prediction on the smallness of ImY(ω), one has to carry out direct measurements by actually transmitting high-frequency signals through atom-sized contacts.

### 3.2. Radio Frequency (RF) Signal Transmission through Atom-Sized Contacts

**Figure 5:**
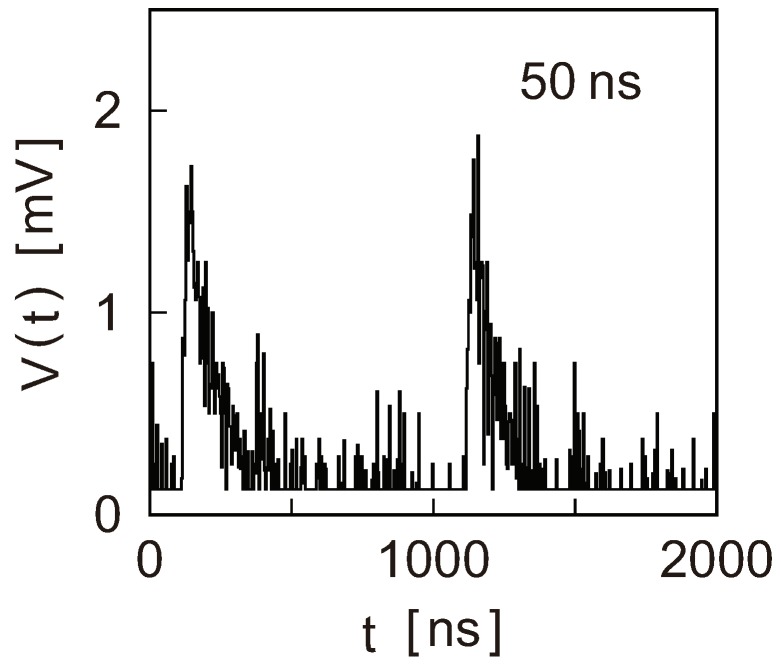
Output pulses from a single-atom contact of Cu when rectangular pulses of 50 ns wide were injected. Because of a large stray capacitance, the pulse shape is heavily distorted from its rectangular waveform. Reproduced from [57], copyright (2010) The Japan Society of Applied Physics.

The RF signal transmission through atom-sized contacts has been first attempted by Mizukami et al. [57]. They used a thin coaxial wire as a specimen of the mechanically controllable break junction (MCBJ) technique [58] and turned the center conductor of the wire into an atom-sized contact of Cu. Employing the junction self-breaking method developed by Tsuitsui et al. [59], a single-atom contact of Cu could be maintained at room temperature for ∼5 s, a sufficiently long time for transmitting and detecting RF signals. Mizukami et al. injected rectangular pulses of various widths and could observe output pulses for pulse widths down to 50 ns. Figure 5 shows the output pulses observed for the 50-ns input pulses. The output pulses are heavily distorted and look like sawtooth or spike signals. Fitting of the output pulse shape indicated a shunt capacitance of ∼40 pF. This should not, however, be an intrinsic capacitor of the Cu single-atom contacts because the same capacitance appeared when the contact was replaced by a fixed resistor. Then, the intrinsic capacitance of the Cu single-atom contacts should be small for 50-ns rectangular pulses or, in terms of spectral bandwidth, for frequencies up to ∼100 MHz.

**Figure 6:**
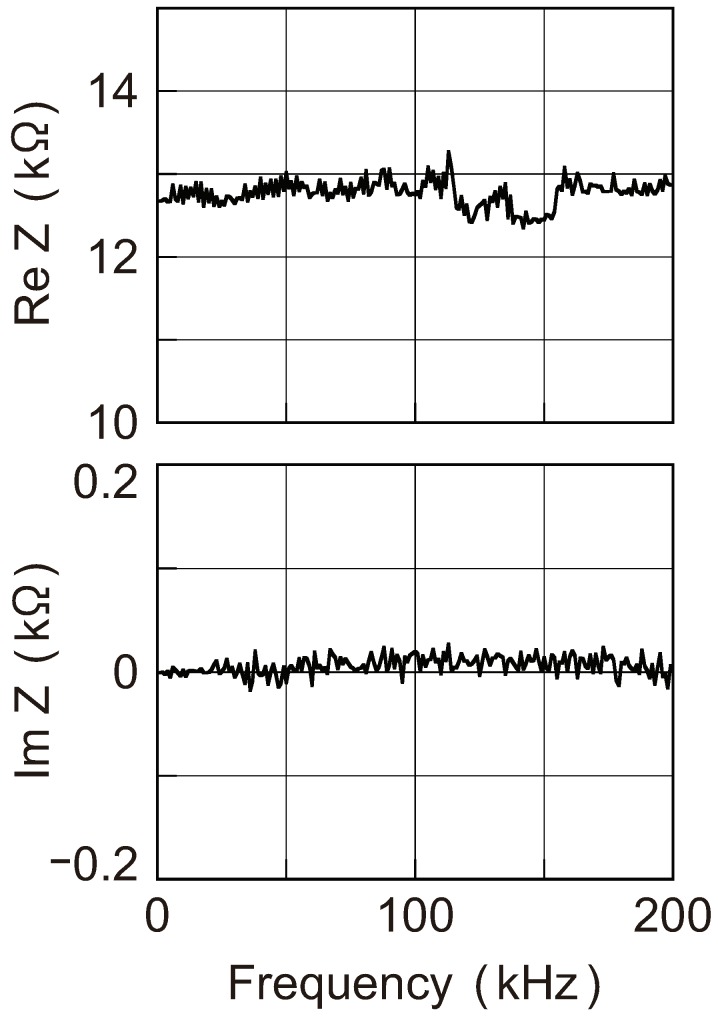
Impedance spectra Z(ω) of a single-atom contact of Au. Upper and lower panels represent ReZ(ω) and ImZ(ω), respectively. Both parts show a flat spectrum up to 200 kHz and ReZ(ω)∼1/G0 and ImZ(ω)∼0. Reproduced from [57], copyright (2010) The Japan Society of Applied Physics.

More direct information on the admittance can be obtained by connecting a specimen to an impedance meter or a network analyzer. The MCBJ technique, combined with the junction self-breaking, enables us to maintain atom-sized contacts for a sufficiently long time during which we can switch the measuring instrument from a DC ammeter to an impedance meter or a network analyzer. Using this technique, the impedance Z(ω)=1/Y(ω) of the Au single-atom contacts has been measured with an impedance meter [57], and the results are depicted in Figure 6. As shown in the figure, ReZ(ω)∼1/G0 and ImZ(ω)∼0 for frequencies up to 200 kHz.

The admittance of atom-sized contacts at higher frequencies was investigated Aoyama et al. [60]. They employed a vector network analyzer and studied the transmission of RF signals through atom-sized contacts of Au and Pt for frequencies up to 1 GHz. They actually measured a *S* parameter $S_{21}=\left|S_{21}\right|e^{i\phi }$ which characterizes the signal transmission through a contact and is related to the admittance as $S_{21}\left(\omega \right)=2Z_{0}Y\left(\omega \right)/\left(1+2Z_{0}Y\left(\omega \right)\right)$ where $Z_{0}=50\;\Omega$ is a cable impedance. For atom-sized contacts, $Z_{0}Y\left(\omega \right)\ll 1$ so that $S_{21}\left(\omega \right)$ is proportional to Y(ω). Specifically, $|S_{21}|$ and ϕ determine the amplitude ratio and the phase difference, respectively, between incident and transmitted signals. The parameters $|S_{21}|$ and ϕ are thus more directly relevant to signal transmission than Y(ω).

**Figure 7:**
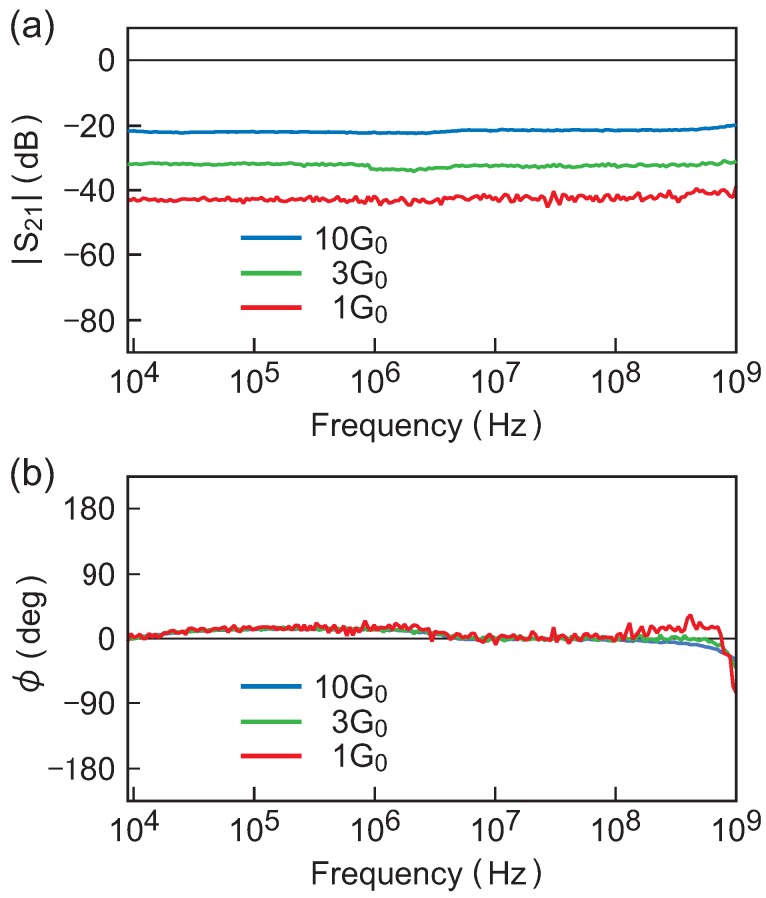
The $S_{21}$ parameter measured on the $10G_{0}$, $3G_{0}$, and $1G_{0}$ contacts of Au and for frequencies up to 1 GHz. Panels (**a**) and (**b**) show the spectrum of $|S_{21}|$ and that of the phase ϕ, respectively. Note that ϕ∼0 over the entire frequency range while $|S_{21}|=\left(constant\right)$ with a constant that varies in proportion to the contact conductance. Reprinted from [60] with the permission of AIP Publishing.

Figure 7a,b show the frequency spectrum of $|S_{21}|$ and ϕ, respectively, obtained for $1G_{0}$, $3G_{0}$, and $10G_{0}$ contacts of Au. A unique advantage of the MCBJ technique is that measurements on different conductance states (i.e., different contact sizes) can be made by slightly varying the substrate bending without causing any changes in the macroscopic experimental configuration. Modifications occur only in an atomic-scale region around the contact, and all sources of stray effects, e.g., wiring to the specimen, are unaffected. Therefore, any spectral features that commonly appear in the data taken on different contacts can be judged as non-intrinsic contributions. In Figure 7b, for example, ϕ exhibits a slight upward bulging around 0.1–1 MHz and a decrement near 1 GHz. These behaviors are commonly observed for $1G_{0}$, $3G_{0}$, and $10G_{0}$ contacts and represent some stray effects. Except these deviations, ϕ∼0 for three contacts over the entire frequency range: the signal passes through these contacts with no phase delay or advance. This leads to ImY(ω)∼0. When ImY(ω) vanishes, Y(ω)=ReY(ω)=τG0 from Equation (4). Then, $|S_{21}|\propto |Y\left(\omega \right)|=\tau G_{0}$ and $|S_{21}|$ becomes a constant. In fact, in Figure 7a, the observed $|S_{21}|$ shows a flat spectrum at different levels depending on the contact conductance. The ratio between these levels is $|S_{21}|\left(1G_{0}\right)$:$|S_{21}|\left(3G_{0}\right)$:|$S_{21}|\left(10G_{0}\right)$ = 1:3.65:13.3 which roughly agrees with the conductance ratio 1:3:10 of three contacts. Thus, both the observed $|S_{21}|$ and ϕ indicate ImY(ω)∼0, as theoretically predicted, and that the atom-sized contacts of metals behave as pure resistors in the RF region.

The experiment by Aoyama et al. [60] also suggests necessary precautions when carrying out MCBJ measurements in the RF region. For example, the connection between a specimen and coaxial wires should be kept as short as possible. One of major stray sources is an insulator-coated metal substrate that produces a large stray capacitance when a metal wire is glued on it; Plastic substrates such as Kapton foils show no such stray effects. However, they cannot be used at cryogenic temperatures, a preferred environment where many atom-sized contacts are more stabilized and longer-lived than at room temperature. Fortunately, the Au atom-sized contacts exhibited superior stability even at room temperature and caused no serious difficulties. In the experiment made by Aoyama et al. [60], they switched the measuring instrument back-and-forth between an ammeter and a network analyzer. This switching failed some contacts, but a reasonable fraction of the tested Au atom-sized contacts was found to survive switching disturbances.

It should be pointed out that the smallness of ImY(ω) has been checked for transparent contacts such as single-atom contacts of Au and Cu but not for others. In the atom-sized contacts of transition metals such as Fe and Ni, multiple conductance channels of low τ contribute to electron transmission [3]. These contacts, therefore, do not fit to the theoretical models described in Section 2 where a single transmission channel is assumed. However, both Equations (5) and (12) predict a small ImY(ω) for a low-τ channel so that ImY(ω) of multiple channel contacts should still remain small if each channel is independent and makes an additive contribution to ImY(ω).

Finally, I note that time-varying biases play an important role not only in signal transmission but also in contact stability. Single-atom metal contacts become destabilized at high biases and exhibit a current-induced breakdown [61]. One of probable failure mechanisms is electromigration (EM). It is well known that device interconnects become longer-lived as the bias frequency increases, due to the healing effect of AC current where migration damages produced during a positive current cycle can be recovered during a next negative cycle [62]. It can therefore be anticipated that single-atom metal contacts would similarly show a longer lifetime under high-frequency biases. For making the healing effect work, the frequency of bias modulation must exceed the thermal hopping frequency of contact atoms which increases to ∼1 MHz when the bias approaches the breakdown voltage [63]. Conventional current-disruption measurements [48,49,50] are difficult to perform at such high frequencies, and it remains an open question whether single-atom contacts are more stabilized under high-frequency biases. The answer to this question will become a critical issue when atom-sized interconnects transmit high-amplitude RF signals.

## 4. Admittance of Molecular Junctions

The theoretical admittance of molecular junctions can be estimated more easily than that of metal contacts because the parameters $E_{0}$ and Γ in the single-level model can be obtained from experiment by fitting a theoretical *I*–*V* curve to an experimental one [27]. Yamauchi et al. [64] applied this method to Au/BDT(1-4 benzenedihiol)/Au junctions and found that $E_{0}$ and Γ lie in energy ranges 0.4–1.0 eV and 20–120 meV, respectively. Even though Au/BDT/Au is known as a highly conductive junction, its conductance is still around $0.01G_{0}$ [64,65]. The Au/BDT/Au is thus a low-transmission junction, and its admittance can be written as $\left(G_{0}/4\right)\left(\hbar \omega /\Gamma \right)\tau$ from Equation (5). By substituting Γ obtained from experimental *I*–*V* curves, the admittance can be estimated as ImY(ω)≲6 pS at ω=1 GHz. As for atom-sized metal contacts, the susceptance of Au/BDT/Au should be negligibly small in the RF region.

Yamauchi et al. [64] also carried out admittance measurements on Au/BDT/Au single-molecule junctions. The observed ImY(ω) started to positively deviate from zero around 20 MHz and, after showing a broad maximum, rapidly decreased to become negative at ≳200 MHz. Yamauchi et al., however, concluded that these variations of ImY(ω) should not be of molecular origin because the ImY(ω) of blank junctions with no bridging molecules showed a similar behavior. Then, the intrinsic ImY(ω) of Au/BDT/Au would remain ∼0 in the RF region in agreement with the theoretical prediction. The observed variations of ImY(ω) would likely be the stray effect generated by the use of metal substrates, as noted in the previous section.

Because the RC time constant of a molecular junction varies as 1/Γ, some molecular junctions showing smaller Γ than that of Au/BDT/Au might start to exhibit a capacitive behavior even in the RF region. However, such low-Γ junctions are almost insulators and of no practical use as device interconnects. In other applications such as single-electron transistors [66], capacitive molecular junctions are indeed a crucial element, but this should be discussed in a different context. As far as the signal transmission is concerned, the results obtained on Au/BDT/Au would be sufficient to indicate that other conductive molecular junctions would also behave as pure resistors in the RF region.

## 5. Conclusions

Theoretical and experimental studies surveyed in the previous sections unanimously lead to a conclusion that ImY(ω)∼0 for atom-sized contacts of metals and for signal frequencies up to 1 GHz. These contacts thus transmit signals with no phase lags when stray effects are minimal and signal bandwidth does not extend to a THz region. Designers of atomic-scale devices will thus be able to safely incorporate atom-sized contacts of metals, including single-atom contacts, into devices as purely resistive elements.
